# The Regulation Network and Clinical Significance of Circular RNAs in Breast Cancer

**DOI:** 10.3389/fonc.2021.691317

**Published:** 2021-07-09

**Authors:** Juan Xu, Xiyi Chen, Yu Sun, Yaqian Shi, Fang Teng, Mingming Lv, Chen Liu, Xuemei Jia

**Affiliations:** ^1^ Deparment of Gynecology, Nanjing Maternity and Child Health Care Hospital, Women’s Hospital of Nanjing Medical University, Nanjing, China; ^2^ Department of Breast, Nanjing Maternity and Child Health Care Hospital, Women’s Hospital of Nanjing Medical University, Nanjing, China; ^3^ Department of Medical Genetics, Nanjing Medical University, Nanjing, China

**Keywords:** circRNA, breast cancer, biogenesis, degradation, distribution, oncogenic, tumor suppressive, biomarker

## Abstract

Breast cancer is one of the most common malignant tumors in women worldwide. Circular RNA (circRNA) is a class of structurally stable non-coding RNA with a covalently closed circular structure. In recent years, with the development of high-throughput RNA sequencing, many circRNAs have been discovered and have proven to be clinically significant in the development and progression of breast cancer. Importantly, several regulators of circRNA biogenesis have been discovered. Here, we systematically summarize recent progress regarding the network of regulation governing the biogenesis, degradation, and distribution of circRNAs, and we comprehensively analyze the functions, mechanisms, and clinical significance of circRNA in breast cancer.

## Introduction

Breast cancer is the most common female malignancy worldwide. According to authoritative cancer statistics in 2021 (1), breast cancer has become the most prevalent of all cancers. An abundance of epidemiological studies has led to the identification of a variety of risk factors for developing breast cancer, including age, family history, early menarche, late pregnancy and menopause, high estrogen level, and excessive dietary fat intake (2).

Breast cancer can be generally divided into four molecular subtypes according to the status of the expression of the joint hormone receptors (estrogen receptors [ER] and progesterone receptors [PR]) and of the human epidermal growth factor receptor 2 (HER2). These subtypes include Luminal A, in which hormone receptors are expressed but HER2 is not (ER+PR+HER2−); Luminal B, in which hormone receptors and HER2 are expressed (ER+PR+HER2+); HER2 overexpression, in which hormone receptors are absent but HER2 is expressed (ER-PR-HER2+); and triple negative breast cancer (TNBC), in which all of the noted receptors are absent (ER-PR-HER2−). Endocrine and targeted therapies can be effective for ER+ or HER2+ breast cancer patients. These treatments include the use of tamoxifen to block the effects of estrogen and trastuzumab to target HER2 receptors on breast cancer cells (3, 4). However, such treatments are ineffective for TNBC, and chemotherapy is regarded as the main systematic treatment for TNBC. Because the efficacy of chemotherapy is unsatisfactory, there exists a desperate need for effective therapies for TNBC (5). Therefore, it is important to explore new treatment regimens to improve current therapeutic strategies, especially for TNBC patients.

One potentially underappreciated class of biological molecules that may yield effective therapeutic targets is circular RNAs (circRNA). CircRNAs generally form single-stranded covalently closed circular structures by the joining of the 3′ and 5′ ends (6). Two key pathways leading to circRNA formation have been proposed in recent authoritative articles. One mechanism involves the backsplicing of exons during transcriptional activities; this splicing is facilitated by base pairing of reverse repeat elements, such as *Alu* elements, located in flanking introns or by RNA binding proteins (RBP). In another key mechanism, circRNAs can be generated from lariat precursors formed in exon-skipping events, as well as from intronic lariat precursors escaping from debranching (7–9). Approximately 80% of circRNAs come from the backsplicing of exons from precursor mRNA or lncRNA (10), but the first circRNA molecules to be discovered, viroids, were found more than 40 years ago to be produced independent of a backsplicing mechanism (11). No matter the mechanism of formation, circRNAs tend to have the following characteristics: (1) CircRNAs are widely and abundantly present in tissues and bodily fluids, and some circRNAs accumulate to a higher level than their respective linear counterparts because of their stable covalently closed structure (9). (2) Many circRNAs are evolutionarily conserved in eukaryotes, in part because they exert critical biological functions (8). (3) Specific circRNAs are typically expressed in a tissue- or cell-specific manner (8, 12).

**Figure 1:**
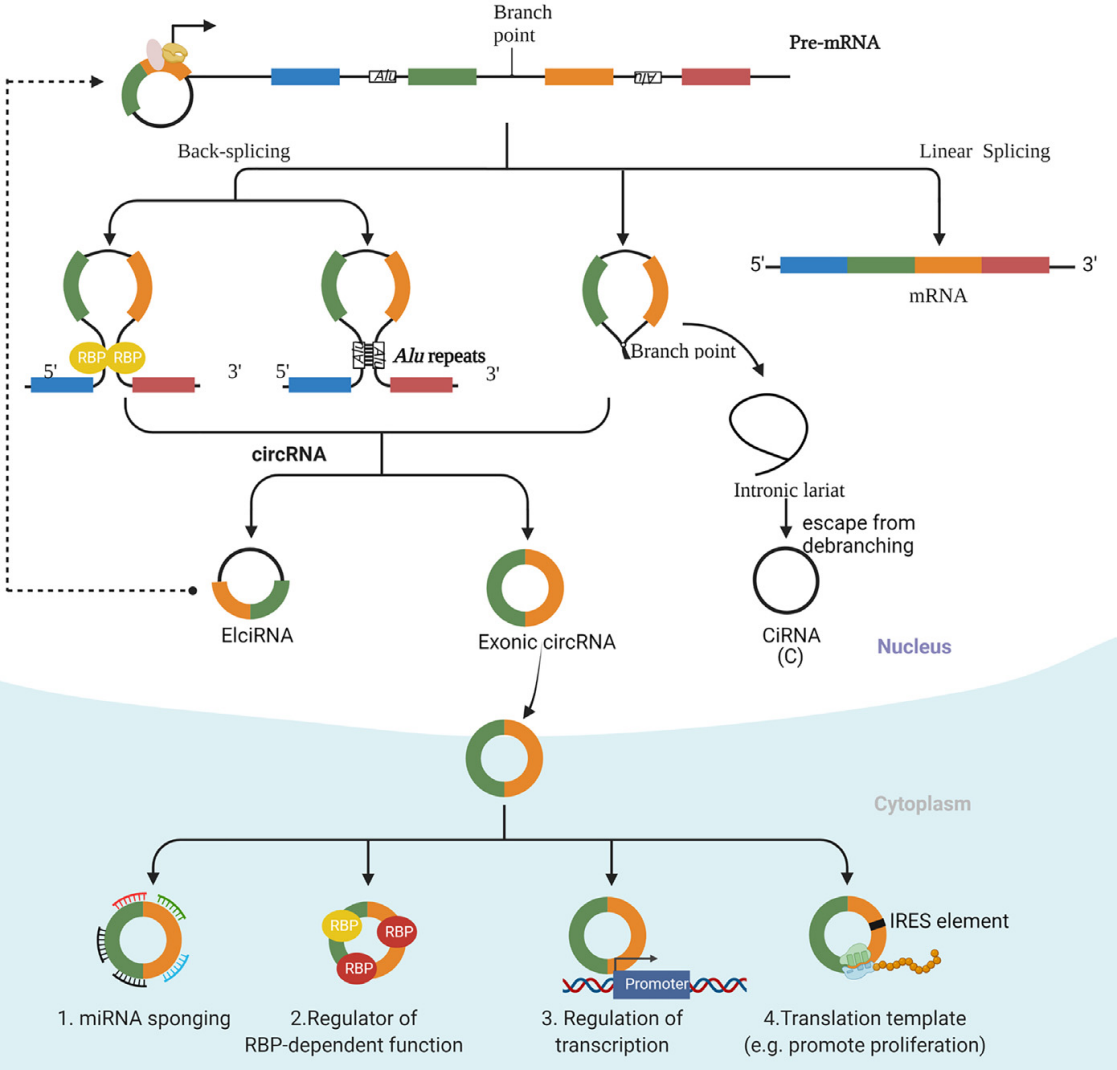
The biogenesis and function of circRNAs. There are three main types of circRNAs, including exonic circRNAs, which consist of only exon(s), exon-intron circRNAs (ElciRNA), which consist of both exon(s) and intron(s), and intronic circRNAs (CiRNA), which consist of only intron(s). An exon-intron circRNA can regulate the expression of its parental gene through binding to its promoter. The mechanism of regulation by exonic circRNAs include serving as a decoy for binding to microRNAs (miRNAs); interacting with specific binding elements on RBPs and acting as a sponge to regulate the functions of RBPs and to affect the activities of associated proteins; regulating transcription; and acting as templates to encode proteins.

Improvements in RNA sequencing technology have led to the identification of several types of circRNAs, including exonic circRNAs, which consist of only exon(s), exon-intron circRNAs (EIciRNA), which consist of both exon(s) and intron(s), and intronic circRNAs (CiRNA), which consist of only intron(s) (**Figure 1**). Cytoplasmic exonic circRNAs tend to have much longer intracellular half-lives compared with their linear counterparts (half-lives of circRNAs average approximately 48 h as opposed to roughly 10 h for many linear RNA molecules). A generally higher intracellular level of circRNAs than their linear counterparts results from this stability, and the stability can be explained at least partly by the resistance of circular RNA molecules to digestion by exonucleases (13, 14).

Other characteristics that are specific to the size and sequence of particular circRNAs have also been discovered among exonic circRNAs. Jeck *et al.* have reported that the length of a given exon appears to influence circularization, a factor that is especially evident for those circles that consist of a single exon (15). Memczak *et al.* has shown that exonic circRNAs described to date generally involve the GT-AG pair of canonical splice sites (16). Moreover, the flanking intron sites that are involved in the backsplicing process generally have relatively long intron sequences, but some exceptions to the lengths of these sequences exist (9). Finally, the sequence of the circRNA can impact cellular localization and activity, as EIciRNA and CiRNA are mainly located in the nucleus and regulate the transcription of specific genes (17, 18).

The most well-established biological function of circRNA involves its serving as a decoy for binding to microRNAs (miRNAs). This activity of circRNA thus modulates the effects of miRNA effects on their target transcripts. In addition, circRNAs can interact with specific binding elements on RBPs and can act as a sponge to indirectly regulate the functions of RBPs and to affect the activities of associated proteins. Finally, circRNAs can recruit specific proteins to certain loci, such as promoter regions, so as to facilitate the transcription of their own host genes (8, 10). Interestingly, under certain circumstances, some circRNAs with internal ribosome entry site (IRES) elements and AUG sites have been reported to be translated into specific peptides, which points to their underestimated cap-independent translational potency (19) (**Figure 1**).

In recent years, regulators of circRNA biogenesis, degradation, and distribution have been identified with increasing rapidity. In addition, various types of circRNA have been found to be abnormally expressed in breast cancer, and their aberrant expression correlates with the occurrence, development, and prognosis of breast cancer; thus, circRNAs may be an important therapeutic target for breast cancer. In this review, we summarize the biogenesis, degradation, and distribution of circRNAs and the mechanisms of action of their regulators. We focus on cutting-edge discoveries concerning functions of circRNAs as well as associated mechanisms and their clinical significance in breast cancer.

## The Regulators of circRNA Biogenesis

### RNA Splicing Factor Regulates the Cyclization and Biogenesis of circRNA

CircRNAs are generally formed by backsplicing of mRNA from canonical splice sites (19). Because RNA splicing factors are central to this process, these factors are important to the regulation of circRNA biosynthesis. For example, Ashwal-Fluss *et al.* discovered that circRNA exon cyclization and canonical pre-mRNA splicing are mutually exclusive and that this competition is a tissue-specific and conservative mechanism of regulation of circRNA in animals (20). They found that a circular RNA related to the muscleblind gene (circMbl) and its flanking introns contain conserved binding sites for the MBL protein. Therefore, the level of MBL present strongly affects circMbl biosynthesis, and the authors confirmed that this effect depends on specific binding sites for MBL (20). Similarly, Pagliarini *et al.* found that SAM68 can bind to the distal intron inverted repeat *Alu* sequence of the spinal muscular atrophy gene (*SMN*), thereby supporting circRNA biosynthesis *in vivo* and *in vitro* (21). Splicing factor proline/glutamine-rich (SFPQ) is specifically enriched in introns flanking a type of Distal-Alu-Long-Intron (DALI) circRNA that is characterized by distal inverted *Alu* elements and long flanking introns, and depletion of SFPQ has been shown to significantly repress DALI circRNA production (22).

The importance of circRNA and its regulation have been demonstrated for several cancer types. Li *et al.* for example, found a global downregulation of circRNA levels in hepatocellular carcinoma. They further demonstrated that inhibition of the RNA splicing factor nudix hydrolase 21 (NUDT21) in this cancer type can promote the formation of circRNA and the production of UGUA sequences. Notably, UGUA sequences are crucial for circRNA formation, and thus this system establishes a positive feedback acceleration of circRNA production (23). In another carcinoma, oral squamous cell carcinoma, Zhao *et al.* found that circUHRF1 could bind to and inhibit miR-526b-5p and thus up-regulate the expression of c-MYC. Overexpression of c-MYC then facilitates the transcription of TGF-β1 and epithelial splicing regulatory protein 1 (ESRP1). Interestingly, ESRP1 itself targets flanking introns of circUHRF1, thereby accelerating circUHRF1 cyclization and biosynthesis (24). In gliomas, Liu *et al.* found that splicing factor serine and arginine-rich splicing factor 10 (SRSF10) can bind to *Alu* sequences of DNA surrounding a circular RNA related to ataxin-a (circATXN1) to regulate biosynthesis of this circRNA. Circ-ATXN1 levels were, therefore, decreased significantly upon knocking down of SRSF10 (25).

### RNA Binding Proteins Regulate the Biogenesis of circRNA

Several other proteins with RNA binding activity have been shown to affect circRNA levels. For example, the immune factor NF90/NF110 contains double-stranded RNA binding domains and can bind to circRNA in the cytoplasm. In addition, however, the production of circRNA has been shown to increase upon association of nuclear NF90/NF110 with intronic RNA pairs flanking circRNA-forming exons. During viral infection, NF90/NF110 translocates from the nucleus to the cytoplasm, leading to a decrease in circRNA formation (26).

These regulatory abilities have, importantly, been shown to be important mediators in several developmental processes. In lung cancer, trinucleotide repeat-containing gene 6A (TNRC6A) can bind to introns near the exons that form circRNA to regulate production of circ0006916 (27). In induced pluripotent stem cell (iPSC) cells, the RNA binding protein fused in sarcoma (FU) can bind to introns near circular RNA reverse splicing sites to regulate circRNA biosynthesis (28). In prostate cancer, P53 can regulate activation of RNA binding motif protein 25 (RBM25), thus affecting the binding of RBM25 to circAMOTL1L. This binding is important for the induction of the biosynthesis of circAMOTL1L (29), because RBM25 mainly binds to the splicing complex, which in turn regulates selective splicing in combination with poly-G or exon splicing enhancer 5′- CGGGCA-3′ sequences (30, 31).

In TNBC, transcription factor E2F1 binds to the promoter of the gene that encodes septin-9 (SEPT9) and promotes the transcription of SEPT9 and the biogenesis of circSEPT9. Another transcription factor, EIF4A3, also regulates the biogenesis of circSETP9 by binding to the upstream and downstream sequences of the circSEPT9 exon (32). In hepatocellular carcinoma, RBM3 binds to SCD-circRNA 2 and regulates its biosynthesis, though the actual mechanisms underlying this regulation are not yet clear (33).

### m^6^A Modification Regulates circRNA Biogenesis and Stability

*Cis* factors are also responsible for the control of biogenesis and stability of circRNAs. A recent study found that backsplicing occurs mostly at sites enriched in N-6-methyladenosine (m^6^A). For example, in male germ cells, approximately half of the circRNA molecules contain an m^6^A-modified initiation codon (34). Similarly, in macrophages of patients with acute coronary syndrome, knockdown of the N^6^-adenosine-methyltransferase METTL3 can down-regulate the m^6^A modification of hsa_circ_0029589 and promote biogenesis of this circRNA (35). Specifically relating to cancer, in hepatocellular carcinoma, m^6^A modification has been found to increase the cellular levels of circ_SORE by enhancing its stability (36).

Di Timoteo *et al.* found that m^6^A modification regulates the accumulation of circ-ZNF609 by regulating the back-splicing of circ-ZNF609, and they found direct correlations among a requirement for the presence of the METTL3 methyltransferase, binding of YTHDC1, and the back-splicing of the m^6^A modified exon (37), suggesting that methylation-mediated regulation might be a general phenomenon in circRNAs. Zhou *et al.* found that the m^6^A reader proteins YTHDF1, YTHDF2, and YTHDC1 bind to m^6^A-modified circRNA and that binding of YTHDF2 in particular reduces the stability of the circRNA (38). Thus, it can be concluded that m^6^A modification sites can function together with accessory proteins, including m^6^A writers, erasers, and readers, to control circRNA biogenesis.

## The Regulation of circRNA Degradation

The levels of circRNA can be controlled at both the synthesis and degradation levels. It has been universally acknowledged that the degradation of most mRNAs initiates with poly(A)-tail shortening at the 3′ end, whereas some mRNAs undergo interior cleavage by endonucleases. Because of the lack of a 5′ 7-methylguanosine cap or a 3′ poly (A) tail, the cleavage of circRNAs, on the other hand, are generally dependent on endonucleases, which initiate degradation internally (39, 40). Cleavage of circRNA CDR1 by endonuclease AGO2 has been shown to be assisted by miR-671. This discovery served as the first evidence that some circRNAs can be degraded by endonucleases in a sequence-dependent manner (41).

**Figure 2:**
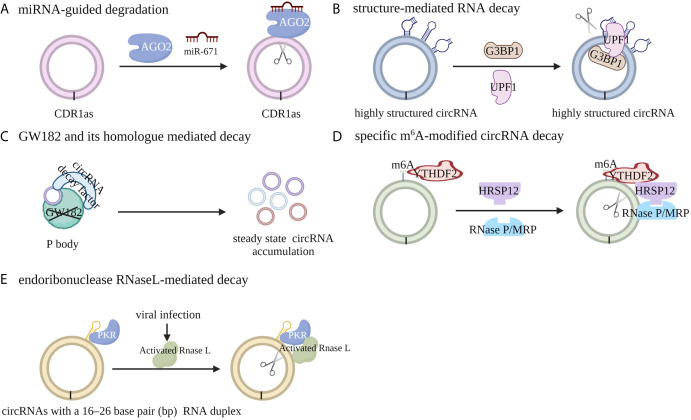
Mechanisms of circRNA degradation. **(A)** CircRNA CDR1 can be cleaved by endonuclease AGO2 with the assistance of miR-671 in a miRNA target sequence-dependent manner. **(B)** The degradation of highly structured circRNAs can be regulated by UPF1 and G3BP1, both of which recognize and unwind the overall structures of circRNAs. **(C)** m^6^A-modified circRNA can recruit the m^6^A reader protein YTHDF2 as well as the adaptor protein HRSP12, and HRSP12 can serve as a bridge to connect YTHDF2 with the endoribonuclease RNase P/MRP, thus enabling degradation of m^6^A modified circRNA by RNase P/MRP. **(D)** The absence of GW182 or its human homologue TRNC6A/B/C, the key component of P body and RNAi complexes, can lead to the accumulation of endogenous circRNA and increase the steady state of cytoplasmic circRNAs. **(E)** Upon viral infection, RNase L can be activated and can degrade circRNAs with RNA duplexes containing 16 to 26 bp through an undefined mechanism.

In general, the pathways leading to circRNA degradation can be divided into five categories: miRNA-guided degradation, structure-mediated RNA decay (SRD), decay mediated by GW182 and its human homolog, specific m^6^A-modified circRNA decay and endoribonuclease RNaseL-mediated decay. Fischer *et al.* reported that the degradation of some highly structured circRNAs can be regulated by UPF1 and G3BP1, both of which recognize and unwind the overall structures of circRNAs (42). The absence of GW182, which is a key component of P body and RNAi complexes, can lead to the accumulation of endogenous circRNA and increase the steady state of cytoplasmic circRNAs, whereas the absence of other factors in the P body or RNAi complex has no similar effect (43). Further study indicated that the MID domain of GW182 protein may mediate the interaction between circRNAs and circRNA decay factors; the absence of TNRC6A, TRNC6B, or TRNC6C, which are the human homologs of GW182, in HEK293 cells, results in the same accumulation of steady-state circRNAs in human cells, indicating a conserved role of P-body and RNAi-mediated degradation of circRNA (43). Park *et al.* reported that m^6^A-modified circRNA can recruit the m^6^A reader protein YTHDF2 as well as the adaptor protein HRSP12, and HRSP12 can serve as the bridge to connect YTHDF2 with the endoribonuclease RNase P/MRP, thus enabling downregulation of m^6^A modified circRNA by RNase P/MRP (44). Liu *et al.* discovered that RNase L, a widely expressed cytoplasmic endoribonuclease, can be activated upon viral infection through an undefined mechanism and degrade the circRNAs with 16 to 26 bp RNA duplex (45) (**Figure 2**).

## Regulators of circRNA Distribution

Once produced, the majority of circRNAs are transported to the cytoplasm where they exhibit further biological functions, whereas some intronic circRNAs and exon-intron circRNAs are thought to reside in the nucleus (17, 18). Since circRNAs lack the classical RNA transport sequence, the nuclear transport mechanism of circRNA remains unclear. As existing studies have indicated that the location of circRNAs determines their functions, it is important to continue to explore the potential mechanisms of circRNA nucleus export.

In order to explore circRNA nuclear transporting mechanisms, Li *et al.* knocked down 26 reported RNA transport-related proteins in *Drosophila*. Their results showed that one circRNA, with a length of 1120 bp, remained in the nucleus, whereas another circRNA, with a length of 490 bp, mainly localized to the cytoplasm after deletion of Hel25E. Both of these circRNAs mainly localize in the cytoplasm in wild-type *Drosophila*. The localizations of 12 other endogenous circRNAs that reside in the cytoplasm in wild-type *Drosophila* were further studied in Hel25E-knockout flies, and it was discovered that circRNAs with lengths of more than 811 bp mainly accumulated in the nucleus, whereas circRNA lengths of less than 702 bp resided in the cytoplasm in cells of the knockout *Drosophila*. An experiment exploring the human Hel25E homologs UAP56 (DDX39B) and URH49 (DDX39A), which share more than 90% similarity with *Drosophila* Hel25E, found that human UAP56 mainly regulates the nuclear transport of long circRNA (more than 1200 bp), whereas URH49 mainly regulates the nuclear export of short circRNA (less than 400 bp). Therefore, the nuclear accumulation of circRNA results mainly from defects of nuclear transport, and the length of circRNA is an important determinant that determines nuclear export (46, 47).

In addition, a very recent study found that m^6^A modification can also influence circRNA nuclear export. It was reported that circNSUN2 could bind to the m^6^A reader YTHDC1, which interacted with the m^6^A binding motif at the GAACU m^6^A within the splice junction of circNSUN2 (48). In studies of mRNA splicing, YTHDC1 has been shown to bind to the RNA splicing factors SRSF1, SRSF3, SRSF7, and SRSF10 to regulate mRNA splicing (49). YTHDC1 can also bind to SRSF3 and the classical nuclear receptor NXF1, thereby regulating the metabolism and transport of m^6^A modified mRNAs (50), suggesting that circRNA may also share the nuclear transporting mechanisms with mRNA.

## CircRNA Profiles in Breast Cancer

By using high-throughput RNA sequencing and microarray analysis, profiles of circRNA expression in breast cancer have been comprehensively analyzed. The RNA sequencing or microarray data of circRNA expression profiles in samples related to breast cancer are listed in **Table 1**. Unlike the studies in gastric cancer and hepatocellular carcinoma that found more down-regulated circRNAs than up-regulated ones (30, 66), in breast cancer, more circRNAs have been found to be up-regulated. These results indicated that the regulation of circRNA levels is tissue specific and that circRNAs likely regulate different process in different systems.

**Table 1:** RNA sequencing and microarray analyses of differentially expressed circRNAs in breast cancer.

Samples	Detection method	Differentially expressed circRNAs	References
4 pairs of TNBC and adjacent noncancerous tissues	RNA sequencing	47 were up-regulated and 307 were down-regulated (FC≥2, *P*< 0.05)	(32)
3 pairs of breast cancer and corresponding adjacent non-cancerous tissues	RNA sequencing	85 were upregulated and 67 were downregulated.(FDR ≤ 0.001, FC≥2)	(51)
8 patients’ specimens (TNBC, *N* = 4; luminal A, *N* = 4) and 3 normal mammary gland tissues (NMGT)	RNA sequencing	140 upregulated and 95 downregulated circRNA were identified, including 215 and 73 circRNAs for the TNBC and LA subtypes, respectively (FC>1.5, *P*<0.05)	(52)
4 TNBC and 3 NMGT	RNA sequencing	122 upregulated and 93 downregulated circRNA were identified (FC>1.5, *P*<0.05)	(52)
4 LA and 3 NMGT	RNA sequencing	55 upregulated and 18 downregulated circRNA were identified (FC>1.5, *P*<0.05)	(52)
3 pairs of breast cancer tissues with or without metastasis	RNA sequencing	A total of 51 circRNAs were differentially expressed (FC>1.5, P< 0.05)	(53)
4 pairs of TNBC tissues and adjacent non-cancerous tissues	RNA sequencing	47 were upregulated, whereas 307 were downregulated (FC>2.0, *P*< 0.05)	(54)
4 pairs of breast cancer lesions and matched adjacent normal-appearing tissues	microarray	715 circRNAs were upregulated and 440 circRNAs were downregulated (FC≥2, *P*<0.05)	(55)
3 pairs of TNBC tissues and matched normal mammalian tissues	microarray	173 circRNAs were up-regulated, and 77 circRNAs were down-regulated (FC≥1.5, *P*<0.05, FDR< 0.05)	(56)
4 pairs of breast cancer tissues and adjacent noncancerous tissues	microarray	A total of 2,587 circRNAs with 1.5 fold of upregulation or downregulation (FC>1.5, *P*<0.05)	(57)
4 pairs of breast cancer tissues and adjacent normal tissues	microarray	15 circRNAs upregulated and 16 circRNAs downregulated (FC>4.0, *P*<0.05)	(58)
5 pairs of breast tumor tissues and matched non-tumor tissues	microarray	A total of 716 circRNAs that were differently expressed (FC>1.5, *P*<0.05)	(59)
3 pairs of breast cancer tissues and their adjacent non-tumor tissues	microarray	1953 circRNAs were upregulated and 1700 circRNAs were downregulated (FC>2.0 and *P*< 0.05)	(60)
3 pairs of TNBC and adjacent normal tissues	RNA sequencing	1307 circRNAs were up-regulated and 3726 circRNAs were down-regulated (FC≥2, *P*< 0.05)	(61)
5 pairs of breast cancer tissues and corresponding nontumorous tissues	RNA sequencing	30 were up-regulated and 19 were down-regulated (FC>2, *P*< 0.001)	(62)
2 pairs of BCLM tissues and primary tumor tissues	microarray	559 were upregulated and 661 were downregulated (FC>2, *P*< 0.05)	(63)
6 pairs of breast cancer/adjacent tissues	microarray	2,375 were upregulated, whereas 1,995 were downregulated (FC>2.0 or <0.5, *P*<0 .05)	(64)
3 pairs of breast cancer tissues and paired noncancerous tissues	microarray	292 were upregulated and 228 were downregulated (FC≥2, P< 0.05)	(65)

BC, breast cancer; LA, luminal A; TNBC, triple-negative breast cancer; NMGT, normal mammary gland tissues; BCLM, breast cancer liver metastases; FC, fold change; FDR, false discovery rate.

## The Diverse Roles of circRNAs in Breast Cancer Progression and Associated Mechanisms

### Mechanisms Leading to Oncogenic Functions of circRNAs in Breast Cancer

Most reported circRNAs have been proposed to function by acting as molecular sponges that bind to and inhibit the functions of miRNA molecules (67, 68). As shown in the RNA sequencing or microarray data, many circRNAs are dysregulated in breast cancer. Among them, the up-regulated circRNAs mainly function as oncogenic factors, whereas the circRNAs down-regulated in breast cancer generally play tumor suppressive roles. The circRNAs that play oncogenic roles in breast cancer are listed in **Table 2**. Here, we discuss several mechanisms through which circRNAs might influence the development or progression of breast cancer.

**Table 2:** CircRNAs that play oncogenic roles in breast cancer.

CircRNA name	Circbase ID	Length	Gene Name	Distribution	Phenotype	Target	Downstream genes/ pathways	Reference
circSEPT9	hsa_circ_ 0005320	645	SEPT6	cytoplasm	promote cell proliferation and migration *in vitro*	miR-637	LIF/STAT3	(32)
circular RNA CDR1as	hsa_circ_0001946	1485	CDR1	\	increase chemosensitivity of 5-FU-resistant BC cells	miR-7	CCNE1	(69)
hsa_circ_0006528	hsa_circ_0006528	496	PRELID2	\	promote adriamycin resistance	miR-7-5p	Raf1	(70)
hsa_circRNA_0006528	hsa_circ_0006528	496	PRELID2	\	promote cell proliferation, invasion, and migration, inhibit apoptosis	miR-7-5p	Raf1-MAPK/ERK	(71)
hsa_circRNA_0000518	hsa_circ_0000518	150	RPPH1	\	and promote cell proliferation, migration, invasion, inhibit cell cycle arrest, apoptosis, *in vitro*, and promote tumor growth *in vivo*	miR-326	FGFR1	(72)
circIQCH	hsa_circ_0104345	864	IQCH	cytoplasm	promote cell proliferation, migration *in vitro*, and promote tumor growth, and metastasis *in vivo*	miR-145	DNMT3A	(73)
circZNF609	hsa_circ_0000615	874	ZNF609	cytoplasm	promote cell proliferation, invasion and migration *in vitro*, and promote tumor growth *in vivo*	miR-145-5p	p70S6K1	(74)
hsa_circ_0136666	hsa_circ_0136666	477	PRKDC	\	promote cell proliferation	miR-1299	CDK6	(75)
circ_0006528	hsa_circ_0006528	496	PRELID2	\	promote proliferation, migration, invasion, autophagy, and inhibit cell apoptosis of PTX-resistant breast cancer cells	miR-1299	CDK8	(76)
circ_0001667	hsa_circ_0001667	456	HEATR2	\	promote cell proliferation, invasion, and migration *in vitro*	miR-125a-5p	TAZ	(77)
hsa_circ_0052112	hsa_circ_0052112	209	ZNF83	cytoplasm	promote cell migration and invasion *in vitro*	miR-125a-5p	ZNF83	(78)
circHMCU	hsa_circ_0000247	792	MCU	cytoplasm	promote cell proliferation, migration, and invasion *in vitro*, and promote tumor growth and lung metastasis *in vivo*	let-7	MCY, HMGA2, and CCND1	(79)
circ-ABCB10	\	\	ABCB10	\	decrease PTX sensitivity and apoptosis, promote cell invasion and autophagy of PTX-resistant BC cells	let-7a-5p	DUSP7	(80)
circPLK1	hsa_circ_0038632	1708	PLK1	cytoplasm	promote cell proliferation, invasion *in vitro*, and tumor occurrence and metastasis *in vivo*	miR-296-5p	PLK1	(81)
hsa_circ_0000515	hsa_circ_0000515	229	RPPH1	cytoplasm	promote cell proliferation, invasion, angiogenesis, and enhance inflammatory response *in vitro*, promote tumor growth *in vivo*	miR-296-5p	CXCL10	(82)
hsa_circ_0011946	hsa_circ_0011946	782	SCMH1	\	promote cell migration and invasion	miR-26a/b	RFC3	(51)
circWWC3	hsa_circ_0089866 and hsa_circ_0001910	1068 and 825	WWC3	cytoplasm	promote cell proliferation, migration, and invasion	miR-26b-3p and miR-660-3p	ZEB1	(83)
circ-DNMT1	hsa_circ_0049224	155	DNMT1	cytoplasm	promote cell proliferation	\	interact with p53 and AUF1	(84)
FECR1	\	571	FLI1	both cytoplasm and nucleus	promote cell invasion *in vitro*	\	binds to the FLI1 promoter *in cis* and recruits TET1, binds to and downregulates DNMT1 *in trans*	(85)
circular HER2	hsa_circ_0007766	676	ERBB2	cytoplasm	promote cell proliferation, invasion, and tumorigenesis *in vitro* and *in vivo*	encoded a novel protein, HER2-103	promote homo/hetero dimerization of EGFR/HER3, sustain AKT phosphorylation	(86)
circIFI30	hsa_circ_0005571	351	IFI30	cytoplasm	promote cell proliferation, migration, invasion, inhibit apoptosis *in vitro*, and promote tumorigenesis and metastasis *in vivo*	miR-520b-3p	CD44	(87)
circAGFG1	hsa_circ_0058514	527	AGFG1	cytoplasm	promote cell proliferation, mobility, and invasion *in vitro*, promote tumorigenesis and metastasis *in vivo*	miR-195-5p	CCNE1	(54)
circKIF4A	hsa_circ_0007255	355	KIF4A	cytoplasm	promote cell proliferation, migration *in vitro*, and promote tumor growth and metastasis *in vivo*	miR-375	KIF4A	(88)
circEPSTI1	hsa_circ_0000479	375	EPSTI1	cytoplasm	promote cell proliferation and inhibit apoptosis	miR-4753 and miR-6809	BCL11A	(56)
circGNB1	hsa_circ_0009362	152	GNB1	cytoplasm	promote cell proliferation, migration *in vitro*, and tumor growth *in vivo*	miR-141-5p	IGF1R	(89)
circ-ABCB10	hsa_circ_0008717	724	ABCB10	\	promote cell proliferation and inhibit apoptosis *in vitro*	miR-1271	\	(57)
hsa_circ_0001982	hsa_circ_0001982	899	RNF111	\	promote cell proliferation, invasion, and inhibit apoptosis	miR-143	\	(59)
circANKS1B	hsa_circ_0007294	459	ANKS1B	cytoplasm	promote cell invasion and migration *in vitro*, promote metastasis *in vivo*	miR-148a-3p and miR-152-3p	USF1-TGF-β1/SMAD	(61)
hsa_circ_0004771	hsa_circ_0004771	203	NRIP1	\	promote tumor growth and inhibit apoptosis	miR-653	ZEB2	(62)
circGFRA1	hsa_circ_0005239	427	GFRA1	cytoplasm	promote cell proliferation and inhibit apoptosis *in vitro*	miR-34a	GFRA1	(90)
circRNA_069718	hsa_circ_0069718	590	DCUN1D4	\	promote cell proliferation, invasion *in vitro*	\	reduce the expression of Wnt/β-catenin pathway-related genes	(91)
circular RNA ciRS-7	hsa_circ_0001946	1485	antisense strand of CDR1	cytoplasm	promote cell migration and invasion *in vitro*	miR-1299	MMPs	(92)
circRNA-CER	hsa_circ_0023404	180	RNF121	\	promote cell proliferation and migration *in vitro*	miR−136	MMP13	(93)
circ-RNF111	hsa_circ_0001982	899	RNF111	\	promote cell proliferation, invasion, glycolysis, and PTX resistance in PTX-resistant BC cells *in vitro*, and enhanced PTX sensitivity *in vivo*	miR-140-5p	E2F3	(94)
circACAP2	\	\	\	cytoplasm	promote cell proliferation and motility	miR-29a/b-3p	COL5A1	(95)
circVAPA	hsa_circ_0006990	338	VAPA	cytoplasm	promote cell proliferation, migration, invasion	miR-130a-5p	\	(96)
hsa_circ_002178	\	\	\	cytoplasm	promote cell proliferation, invasion, and migration	miR-1258	KDM7A	(97)
hsa-circ-0083373, hsa-circ-0083374, hsa-circ-0083375	hsa_circ_0083373, hsa_circ_0083374, hsa_circ_0083375	3868,4045,5469	DLC1	\	promote pathogenesis and development of breast cancer	hsa-miR-511	\	(98)
circRNA_100876	\	\	\	\	promote cell proliferation and invasion	miR-4753 and miR-6809	BCL11A	(99)
circ_0103552	hsa_circ_0103552	920	UBR1	\	promote cell proliferation, migration, invasion, and inhibit apoptosis	miR-1236	\	(100)
circMMP11	hsa_circ_0062558	906	MMP11	cytoplasm	promote cell proliferation and migration *in vitro*	miR-1204	MMP11	(101)
circ-UBE2D2	\	\	UBE2D2	\	promote cell proliferation, migration, and invasion *in vitro*	miR-1236 and miR-1287	\	(102)
circ_UBE2D2	hsa_circ_0005728	280	UBE2D2	cytoplasm	promote tamoxifen resistance	miR-200a-3p	\	(103)
circUBE2D2	hsa_circ_0005728	280	UBE2D2	cytoplasm	promote cell proliferation, migration, and invasion, and doxorubicin resistance *in vitro*, promote tumor growth *in vivo*	miR-512-3p	CDCA3	(104)
hsa_circ_0091074	hsa_circ_0091074	373	TCONS_00016926	\	promote cell proliferation and invasion	miR−1297	TAZ/TEAD4	(105)
circ_0000043	hsa_circ_0000043	438	PUM1	\	promote cell proliferation, migration, invasion, and EMT	miR-136	SMAD3	(106)
hsa_circ_0003645	hsa_circ_0003645	356	C16orf62	cytoplasm	promote cell proliferation and inhibit cell apoptosis *in vitro* and *in vivo*	miR-139-3p	HMGB1	(107)
circCDYL	hsa_circ_0008285	667	CDYL	cytoplasm	promote the malignant progression *in vitro* and *in vivo*	miR-1275	ATG7 and ULK1	(108)
circ_0000520	hsa_circ_0000520	123	RPPH1	cytoplasm	promote cell proliferation, migration, and invasion, but inhibit cell cycle arrest and apoptosis	miR-1296	SP1	(109)
circular RNA KIF4A	hsa_circ_0007255	355	KIF4A	cytoplasm	promote cell migration, invasion, and inhibit apoptosis	miR-152	ZEB	(110)
circABCC4	hsa_circ_0030586	1192	ABCC4	\	promote cell proliferation, migration, invasion, and inhibit apoptosis	miR-154-5p	promote NF-κB and WNT/β-catenin pathways	(111)
circDENND4C	\	\	DENND4C	\	increase glycolysis, migration, and invasion under hypoxia	miR-200b and miR-200c	MMP2, MMP9	(112)
circDENND4C	\	\	\	\	promote cell migration and invasion under hypoxia *in vitro*, promote tumor growt*h in vivo*	\	\	(113)
circHIPK3	hsa_circ_0000284	1099	HIPK3	cytoplasm	promote cell proliferation, migration, and invasion *in vitro* and tumor growth *in vivo*	miR-193a	HMGB1/PI3K/AKT	(114)
hsa_circ_001783	\	34460	EBLN3- ZCCHC7	cytoplasm	promote cell proliferation and invasion	miR-200c-3p	\	(115)
circABCB10	hsa_circ_ 0008717	724	ABCB10	\	promote cell proliferation, glycolysis, and increase IR resistance	miR-223-3p	PFN2	(116)
circular RNA PVT1	hsa_circ_0001821	410	PVT1	cytoplasm	promote tumor growth *in vivo*	miR-204-5p	upregulate E-cadherin, downregulate N-cadherin, Vimentin, Slug, and Twist	(117)
circRAD18	hsa_circ_0002453	756	RAD18	cytoplasm	promote cell proliferation and migration, inhibit cell apoptosis *in vitro*, promote tumor growth *in vivo*	miR-208a and miR-3164	IGF1 and FGF2	(118)
circRAD18	hsa_circ_0002453	756	RAD18	cytoplasm	promote cell proliferation, migration, invasion, EMT, and inhibit cell apoptosis	miR-613	HK2	(119)
hsa_circ_0131242	hsa_circ_0131242	12400	MAP3K4	\	promote cell proliferation and migration *in vitro*	miR-2682	\	(120)
*hsa_circ_0072995	hsa_circ_0072995	435	ARHGEF28	both nucleus and cytoplasm	promote cell migration and invasion	miR-30c-2-3p	ARHGEF28	(121)
circ-TFF1	hsa_circ_0061825	492	TFF1	cytoplasm	promote cell proliferation, migration, invasion, EMT *in vitro*, and tumor growth *in vivo*	miR-326	TFF1	(122)
circ_0000291	hsa_circ_0000291	311	CD44	\	promote cell proliferation, migration, and invasion	miR-326	ETS1	(123)
circular RNA 0007255	hsa_circ_0007255	355	KIF4A	cytoplasm	promote oxygen consumption, colony formation, and cell mobility *in vitro*, inhibit tumor growth *in vivo*	miR-335-5p	SIX2	(124)
circEIF3M	hsa_circ_0003119	361	EIF3M	cytoplasm	promote cell proliferation, migration, and invasion	miR-33a	cyclin D1	(125)
hsa_circRNA_002178	hsa_circ_0000519	98	RPPH1	cytoplasm	promote cell proliferation, energy metabolism, and angiogenesis	miR-328-3p	COL1A1	(126)
circ-PGAP3	hsa_circ_0106800	537	PGAP3	cytoplasm	promote cell proliferation and invasion	miR-330-3p	Myc	(127)
circIRAK3	hsa_circ_0005505	754	IRAK3	cytoplasm	promote cell migration and invasion *in vitro*, promote metastasis and *in vivo*	miR-3607	FOXC1	(128)
hsa_circ_0008039	hsa_circ_0008039	462	PRKAR1B	cytoplasm	promote cell proliferation and migration *in vitro*	miR-432-5p	E2F3	(129)
hsa_circ_0008039	hsa_circ_0008039	462	PRKAR1B	\	promote cell proliferation, migration, and invasion *in vitro*, and promote tumor growth *in vivo*	miR-515-5p	CBX4	(130)
circular RNA circ-ZEB1	\	\	\	\	promote cell proliferation and inhibit apoptosis	miR-448	eEF2K	(131)
circular RNA-100219	hsa_circ_0004619	377	FAF1	\	promote cell proliferation and migration *in vitro*	miR-485-3p	NTRK3	(132)
circMYO9B	hsa_circ_0000907	898	MYO9B	\	promote cell proliferation, migration, and invasion *in vitro*, and promote tumor growth *in vivo*	miR-4316	FOXP4	(133)
circRNF20	hsa_circ_0087784	499	RNF20	cytoplasm	promote cell proliferation, inhibit cell apoptosis *in vitro*, promote tumor growth *in vivo*	miR-487a	HIF1α-HK2	(134)
hsa_circ_0007534	hsa_circ_0007534	400	DDX42	\	promote cell proliferation, invasion, and inhibit apoptosis *in vitro*	miR-593	MUC19	(135)
circ_DCAF6	\	\	\	cytoplasm	promote cell proliferation and stemness	miR-616-3p	GLI1	(136)
circ_0005230	hsa_circ_0005230	3958	DNM3OS	\	promote cell invasion, migration *in vitro*, and promote cell growth *in vivo*	miR-618	CBX8	(137)
circZFR	hsa_circ_0072088	693	ZFR	\	promote cell proliferation, migration, invasion, glycolysis, and inhibit cell apoptosis *in vitro*, promote tumor growth *in vivo*	miR-578	HIF1A	(138)
circ-UBAP2	hsa_circ_0001846	747	UBAP2	cytoplasm	promote cell proliferation, migrate, and inhibit cell apoptosis *in vitro*, and promote tumor growth and metastasis *in vivo*	miR-66	MTA1	(139)
circFBXL5	hsa_circ_0125597	912	FBXL5	cytoplasm	promote cell proliferation and migration *in vitro*, promote tumor growth and metastasis *in vivo*	miR-660	SRSF6	(140)
circTP63	\	295	TP63	cytoplasm	promote cell proliferation, invasion, migration *in vitro*, promote tumor growth *in vivo*	miR-873-3p	FOXM1	(141)
hsa_circRPPH1_015	hsa_circ_0000517	88	RPPH1	cytoplasm	promote cell proliferation and aggressiveness *in vitro*, promote tumor growth *in vivo*	miR-326	ELK1	(142)
#circANKRD12	hsa_circ_0046841 or \	286 or 925	ANKRD12	cytoplasm	promote cell proliferation, invasion, migration, and alter cell metabolism	\	CCND1	(143)
circCNOT2	\	\	\	\	increase cell proliferation	\	\	(144)
circAMOTL1	\	\	\	\	promote cell proliferation and invasion, and inhibit cell apoptosis when exposed to PAX *in vitro*	\	AKT	(145)
circ_103809	\	\	\	\	promote cell proliferation	\	PI3K/AKT signaling	(146)
hsa_circ_0008673	hsa_circ_0008673	689	BRCA1	\	promote cell proliferation and migration	\	\	(147)
hsa_circ_001569	\	\	\	\	promote cell proliferation, migration, invasion, and inhibit cell apoptosis *in vitro*	\	PI3K/AKT signaling	(148)
circBACH2	hsa_circ_0001627	2995	BACH2	cytoplasm	promote cell proliferation, invasion, and migration *in vitro*, promote tumor growth and metastasis *in vivo*	miR-186-5p/miR-548c-3p	CXCR4	(149)
circPGR	\	\	PGR	cytoplasm	promote ER-positive breast cancer cell proliferation, invasion, and migration *in vitro*, promote tumor growth *in vivo*	miR-301a-5p	Cell cycle genes	(150)

EMT, epithelial mesenchymal transition; PAX, paclitaxel; *The circRNA can simultaneously regulate an miRNA and genes, but the relationship between miRNA and genes is not clear. #Two circular isoforms of 286 bp (hsa_circ_0046841) and 925 bp (not found in the circbase) lengths share the same backsplice junction of ANKRD12, and the study did not distinguish these two circular isoforms.

### Acting as Competing Endogenous RNAs (ceRNAs)

A major mechanism that explains impacts of circRNA levels on cancer development involves the interaction of these RNA molecules with miRNA molecules. This binding event, called “sponging,” can inhibit the interactions of these miRNA molecules with other cellular molecules, frequently leading to inhibition of the miRNA activity. Thus, circRNAs can serve as ceRNAs. The downstream effect of the competition depends on the ultimate target of the miRNA itself.

Multiple circRNAs can target the same miRNA and thus assist malignant progression of breast cancer. For example, hsa_circ_0006528 and circCDR1as can target CCNE1 and RAF1, respectively, through sponging miR-7. In this way, both of these circRNA molecules promote the proliferation, invasion, migration, and chemoresistance of breast cancer cells (69, 70). Similarly, hsa_circ_0000291, hsa_circRNA_000518, and circ_TFF1 can target ETS1, FGFR1, and TFF1, respectively, through sponging miR-326, to accelerate the proliferation, colony formation, invasion, and migration of breast cancer cells; these activities also serve to reduce cell apoptosis (71, 72, 151). CircZNF609 and circIQCH can target DNMT3A and p70S6K1 by sponging miR-145 to promote the malignant phenotype of breast cancer cells (73, 74). Hsa_circ_0136666 and hsa_circ_0006528 can target CDK6 and CDK8 by sponging miR-1299, respectively, to regulate cell cycle progression, proliferation, apoptosis, invasion, migration, and drug resistance of breast cancer cells (75, 76). Hsa_circ_0052112 and hsa_circ_0001667 mainly regulates ZNF36 and FOXO3a by sponging target miR-125a-5p, respectively, to regulate the invasion and migration of breast cancer cells (77, 78). Circ-ABCB10 and circHMCU can sponge let-7 then regulate the expression of MCY, HMGA2, CCND1, and DUSP7, respectively, to promote the proliferation, invasion, migration, and chemoresistance of breast cancer cells (79, 80). CircPLK1 and hsa_circ_0000515 can target miR-296-5p to regulate the expression of PLK1 and CXCL10, respectively, to promote the growth, proliferation, migration and inflammatory response of breast cancer cells (81, 82). And surprisingly, hsa_circ_0011946 can interact with miR-26a/b to regulate RFC3, to promote the invasion and migration of breast cancer cells, whereas circWWC3 can interact with miR-26b-3p and miR-660-3p to regulate the expression of ZEB1, and thus promote the proliferation, invasion and migration of breast cancer cells (51, 83).

It is important to note the potential for ceRNA-acting circRNA molecules to improve patient outcomes in addition to their roles in cancer progression. For example, TV-circRGPD6 can interact with miR-26b. This interaction serves to regulate the expression of YAF2 and thus leads to a better prognosis (152).

#### Protein Decoy or Scaffolding Functions

In some cases, circRNAs may modulate the progression of breast cancer through direct or indirect interactions with RBPs. These interactions may alter the functions of the RBPs either through competition, with the circRNA serving as a decoy, or through the recruitment of other interacting factors, with the circRNA serving a scaffolding role. For example, in breast cancer cells with wild-type p53, circ-CCNB1 can interact with both p53 and H2AX, and Bclaf1 is free to bind to Bcl2. In p53-mutant breast cancer cells, on the other hand, H2AX is not able to interact with p53, and circ-CCNB1 is thus free to form a complex with H2AX and Bclaf1 and to thus to slow the development of p53 mutation-induced breast cancer progression (153). Similarly, circ-Dnmt1 can interact with p53 and AUF1 to facilitate their nuclear translocation, and this nuclear translocation of p53 induces autophagy. The nuclear translocation of AUF1 can increase mRNA stability and protein expression of Dnmt1, whereas the nuclear translocation of Dnmt1 further inhibits p53 transcription (84). In addition, CircSKA3 can interact with Tks5 and integrin-β1 to promote the invadopodium formation, thereby promoting breast cancer invasion (154).

#### Transcriptional Regulation

In at least one important case, circRNA has been shown to impact cellular biological functions *via* a direct impact on transcription in breast cancer. Specifically, circRNA FECR1 (a circRNA consisting of exons 2, 3, and 4 of the Friend leukemia integration 1 (*FLI1*) gene) has been shown to bind to the promoter of the gene encoding DNA methyltransferase 1 (*DNMT1*) and to down-regulate DNMT1 transcription. Similarly, circRNA FECR1 has been shown to recruit the methylcytosine dioxygenase TET1 to the promoter of FLI1 and thus to induce demethylation at the FLI1 promoter (85).

#### Encoding of Functional Peptides

One circRNA that is related to the growth factor receptor HER2 (circHER2) encodes a 103- amino acid peptide known HER2-103. This peptide promotes the binding of EGFR with HER2 and increases EGFR kinase activity. This activity has been shown to correlate with increased proliferation and invasion of breast cancer cells and to enhance sensitivity to pertuzumab (86).

### Mechanisms Leading to Tumor Suppressive Functions of circRNAs in Breast Cancer

Consistent with the smaller number of circRNAs that are down-regulated in breast cancer, the number of known tumor suppressive circRNAs is much fewer than the oncogenic circRNAs in breast cancer. Similar to the mechanisms leading to enhancement of tumor development, these circRNAs may act as ceRNAs, protein decoys or scaffolds as well as encode functional peptides to suppress breast cancer progression. The known tumor suppressive circRNAs are listed in **Table 3**.

**Table 3:** CircRNAs with tumor suppressive functions in breast cancer.

CircRNA name	CircBase ID	Length	Gene	Distribution	Phenotype	Target	Downstream genes/ pathways	Reference
TV-circRGPD6	\	\	\	\	inhibit tumor-initiating properties *in vitro*, inhibit tumor growth and metastasis *in vivo*	miR-26b	YAF2	(152)
circ-Ccnb1	hsa_circ_0072758	342	CCNB1	nucleus	inhibit cell proliferation, increase cell apoptosis *in vitro*, inhibit tumor growth and extend mouse viability *in vivo*	\	interact with H2AX, p53, and BCLAF1	(153)
circular RNA 000554	hsa_circ_0000376	48782	PRH1-PRR4	cytoplasm	inhibit cell invasion and migration *in vitro*, inhibit tumor growth *in vivo*	miR-182	ZFP36	(155)
circRNA_0025202	hsa_circ_0025202	495	GAPDH	cytoplasm	inhibit cell proliferation, migration, increase cell apoptosis and sensitivity to tamoxifen in HR(+) BC cells *in vitro*	miR-182-5p	FOXO3a	(156)
circular RNA-0001283	hsa_circ_0001283	1400	WDR48	cytoplasm	inhibit cell proliferation, invasion, and promote cell apoptosis	miR-187	HIPK3	(157)
circTADA2A-E6 and circTADA2A-E5/E6	hsa_circ_0006220 hsa_circ_0043278	158 and 250	TADA2A	cytoplasm	inhibit cell proliferation, migration, invasion *in vitro*	miR-203a-3p	SOCS	(52)
circASS1	hsa_circ_0089105	241	ASS1	both nucleus and cytoplasm(majority)	inhibit cell invasion and migration *in vitro*	miR-4443	ASS1	(158)
circKDM4C	hsa_circ_0001839	292	KDM4C	cytoplasm	inhibit cell proliferation, invasion, migration, doxorubicin resistance, promote cell apoptosis *in vitro*, inhibit tumor growth and metastasis, *in vivo*	miR-548p	PBLD	(53)
circBMPR2	hsa_circ_0003218	342	BMPR2	cytoplasm	inhibit cell proliferation, migration, and invasion and tamoxifen resistance	miR-553	USP4	(159)
circular RNA 0001073	hsa_circ_0001073	473	ACVR2A	both nucleus and cytoplasm	inhibit proliferation, migration, invasion, and promote apoptosis *in vitro*, inhibit tumor growth *in vivo*	\	HuR	(160)
circ-Foxo3	\	\	FOXO3	\	inhibit tumor growth and extend mouse lifespan *in vivo*	\	increase Foxo3 protein level but decreasep53 level	(161)
circular RNA−MTO1	hsa_circ_0007874	318	MTO1	both nucleus and cytoplasm	inhibit cell proliferation, promote monastrol-induced cell cytotoxicity and reverse monastrol resistance *in vitro*	\	TRAF4/Eg5	(162)
circFBXW7	hsa_circ_0001451	1227	FBXW7	cytoplasm	inhibit cell proliferation, migration *in vitro* and inhibit tumor growth and lung metastasis *in vivo*	miR-197-3p and FBXW7-185aa	FBXW7	(163)
circRNA-000911	\	\	\	\	inhibit cell proliferation, migration, and invasion, and promote cell apoptosis	miR−449a	NOTCH1/NF-κB	(59)
circEHMT1	\	\	EHMT1	\	inhibit cell migration and invasion *in vitro* and inhibit lung metastasis *in vivo*	miR-1233-3p	KLF4/MMP2	(164)
circNFIC	hsa_circ_0002018	311	NFIC	cytoplasm	inhibit cell proliferation and migration *in vitro*, inhibit cell growth and lung metastasis *in vivo*	miR-658	UPK1A	(165)
circ_0000442	hsa_circ_0000442	7273	MED13L	\	inhibit cell proliferation *in vitro* and inhibit tumor growth *in vivo*	miR-148b-3p	PTEN/PI3K/AKT	(166)
circCDYL	hsa_circ_0008285	667	CDYL	cytoplasm	inhibit cell proliferation, migration, invasion, and promote cell apoptosis	miR-190a-3p	TP53INP1	(167)
circ-ITCH	\	\	ITCH	\	inhibit cell proliferation, migration, and invasion *in vitro*, and inhibit tumor growth and metastasis *in vivo*	miR-214 and miR-17	ITCH	(168)
Circular RNA BARD1	hsa_circ_0001098	1204	BARD1	\	inhibit cell proliferation and promote cell apoptosis *in vitro*, inhibit tumor growth and metastasis *in vivo*	miR-3942-3p	BARD1	(169)
circAHNAK1	hsa_circ_0000320	384	AHNAK1	cytoplasm	inhibit cell proliferation, migration, and invasion *in vitro*, inhibit tumor growth and metastasis *in vivo*	miR-421	RASA1	(170)
hsa_circ_0072309	hsa_ circ_0072309	580	LIFR	cytoplasm	inhibit cell proliferation, migration, and invasion *in vitro*, promote tumor growth *in vivo*	miR-492	\	(171)
circRNA_103809	\	\	ZFR	\	inhibit cell proliferation, invasion, and migration, EMT *in vitro*	miR-532-3p	\	(172)
circDDX17	hsa_circ_0002211	927	DDX17	cytoplasm	inhibit cell proliferation and promote cell apoptosis	miR-605	CDK1 and p21	(173)
hsa_circ_0068033	hsa_circ_0068033	900	NAALADL2	cytoplasm	inhibit cell proliferation, invasion, migration, and promote apoptosis *in vitro*, inhibit tumor growth *in vivo*	miR-659	\	(174)
hsa_circ_0001785	hsa_circ_0001785	467	ELP3	cytoplasm	inhibit cell proliferation, migration, and invasion *in vitro*, inhibit tumor growth *in vivo*	miR-942	SOCS3	(175)
circSMARCA5	hsa_circ_0001445	269	SMARCA5	nucleus	increase cisplatin sensitivity *in vitro* and *in vivo*	\	SMARCA5	(176)
circSCYL2	hsa_circ_0006258	508	SCYL2	\	inhibit cell migration and invasion	\	EMT	(177)
circ-LARP4	\	\	LARP4	\	increase the doxorubicin sensitivity *in vitro*	\	\	(178)
circular RNA VRK1	hsa_circ_0141206	852	VRK1	\	inhibit cell proliferation, promote cell apoptosis *in vitro*	\	\	(179)
circular RNA VRK1	hsa_circ_0141206	852	VRK1	cytoplasm	inhibit cell proliferation, promote cell apoptosis, and inhibit self-renewal capacity *in vitro*	\	\	(180)
circUSP42	hsa_circ_0007823	451	USP42	\	inhibit cell invasion and migration	miR-4443	ASS1	(181)

#### ceRNAs

Both CircRNAs 000554 and circRNA_0025202 can sponge miR-182 to regulate the expression of ZNF36 and FOXO3a, respectively, and thus inhibit cellular proliferation, invasion, migration, and sensitivity to chemotherapy agents (155, 156). Similarly, circRNA-0001283 functions to sponge miR-187 to suppress HIPK3 expression, thus inhibiting the proliferation and invasion and promoting the apoptosis of breast cancer cells (157). CircTADA2A-E6 mainly regulates suppressor of cytokine signaling (SOCS) expression by sponging miR-203a-3p, and this sponging effect correlates with inhibition of breast cancer proliferation, invasion, and migration (52). CircASS1 mainly inhibits breast cancer invasion and migration by sponging miR-4443; it has also been found that the expression of circASS1 is inversely related with that of its parental gene arginosuccinate synthase 1 (ASS1), which indicates that the splicing of ASS1 mRNA and circASS1 may compete with each other (158). CircKDM4C mainly regulates the expression of phenazine biosynthesis like protein domain containing (PBLD) by sponging miR-548p, thereby inhibiting breast cancer growth, metastasis, and drug resistance (53). CircBMPR2 regulates ubiquitin specific peptidase 4 (USP4) expression by sponging miR-553, to inhibit breast cancer proliferation, invasion, migration, and tamoxifen resistance (159).

#### Protein Decoys or Scaffolds

Some tumor suppressive circRNAs function through interacting with proteins. For example, circ-Ccnb1 interacts with p53, inhibits tumor growth and increase the survival time of mice (153). Yi *et al.* reported the direct interaction between circRNA 0001073 and the RNA binding protein HuR (160). Experiments with the apoptosis-related protein receptor-interacting protein (RIP) revealed that the tumor suppressive circRNA circ-FOXO3, which is enriched in normal cells, can interact with MDM2, p53, and FOXO3, facilitating the formation of the MDM2-P53 complex while inhibiting MDM2-FOXO3 binding. This results in promotion of p53 ubiquitination and inhibition of FOXO3 degradation and thus the induction of apoptosis of breast cancer cells (161). Circular RNA-MTO1 can block the interaction between TRAF4 and Eg5, and suppress the translation of Eg5, to inhibit breast cancer growth and reverse the resistance of breast cancer cells to monosterol (162).

#### Encoding of Functional Peptides

circFBXW7 not only can interact with miR-197-3p, but also encodes a 185-amino-acid peptide that regulates the expression of FBXW7 and the degradation of c-Myc, thus inhibiting proliferation and migration of ovarian cancer cells (163).

## Analysis of CircRNAs in the Diagnosis and Prognosis of Breast Cancer

Although circRNAs in plasma are not as stable as those within cells, circRNAs are still considered important diagnostic and prognostic biomarkers for cancer. CircRNAs that are particularly important diagnostic or prognostic biomarkers in breast cancer are listed in **Table 4**.

**Table 4:** CircRNAs with diagnostic and prognostic value in breast cancer.

CircRNA name	Circbase ID	Clinicopathological Association	Tumor type (Sample number)	Reference
circSEPT9	hsa_circ_0005320	TNM stage, T stage, N stage, OS, diagnosis	TNBC (cohort2: n=80; cohort1: n=60)	(32)
circZNF609	hsa_circ_0000615	lymph node metastasis, TNM stage, OS	(n=143)	(74)
circHMCU	hsa_circ_0000247	lymph node metastasis, T stage, N stage, histological grade, OS	(n=267)	(79)
circPLK1	hsa_circ_0038632	tumor size, lymph node metastasis, TNM stage, DFS, OS	TNBC (n=240)	(81)
hsa_circ_0000515	hsa_circ_0000515	OS	(n=340)	(82)
circWWC3	hsa_circ_0089866 and hsa_circ_0001910	clinical stage, OS	(n=156)	(83)
circRGPD6	\	DFS, OS	(n=165)	(152)
circular HER2	hsa_circ_0007766	OS	TNBC (n=59)	(86)
circRNA_0025202	hsa_circ_0025202	lymph node metastasis, histological grade	HR positive breast cancer (n=230)	(156)
circular RNA 0001073	hsa_circ_0001073	tumor size, distant metastasis, TNM stage, RFS, diagnosis	(n=132)	(160)
circFBXW7	hsa_circ_0001451	tumor size, lymph node metastasis, DFS, OS	TNBC (n=473)	(163)
circular RNA VRK1	hsa_circ_0141206	tumor size, TNM stage, T stage, N stage, histological grade, OS, diagnosis	(n=350)	(179)
*circIFI30	hsa_circ_0005571	clinical stage, histological grade, OS, diagnosis	TNBC (n=78/n=38)	(87)
circAGFG1	hsa_circ_0058514	TNM stage, T stage, N stage, OS, diagnosis	TNBC (n=40)	(54)
hsa_circ_006054, hsa_circ_100219, and hsa_circ_406697	\	diagnosis	(n=51)	(55)
hsa_circ_0001785	hsa_circ_0001785	distant metastasis, TNM stage, histological grade, diagnosis	(n=57)	(182)
circKIF4A	hsa_circ_0007255	tumor size, lymph node metastasis, TNM stage, DFS, OS	TNBC (n=240)	(88)
circEPSTI1	hsa_circ_0000479	tumor size, lymph node metastasis, TNM stage, DFS, OS	TNBC (n=240)	(56)
circGNB1	hsa_circ_0009362	tumor size, TNM stage, DFS, OS	TNBC (n=222)	(89)
circANKS1B	hsa_circ_0007294	lymph node metastasis, clinical stage, OS	TNBC (n=165)	(61)
hsa_circ_0006220	hsa_circ_0006220	lymph node metastasis, pathological type, diagnosis	(n=50)	(65)
circGFRA1	hsa_circ_0005239	tumor size, lymph node metastasis, TNM stage, DFS, OS	TNBC (n=222)	(90)
circRNA_069718	hsa_circ_0069718	lymph node metastasis, TNM stage, OS	TNBC (n=35)	(91)
hsa_circ_002178	\	tumor size, lymph node metastasis, OS	(n=83)	(97)
circRNA_100876	\	OS	(n=50)	(99)
circMMP11	hsa_circ_0062558	lymph node, metastasis, TNM stage	(n=113)	(101)
circUBE2D2	\	tumor size, lymph node metastasis, TNM stage, OS, PFS	(n=80)	(102)
circUBE2D2	hsa_circ_0005728	lymph node metastasis, TNM stage, OS	TNBC (n=66)	(104)
circCDYL	\	DFS, OS	(n=113)	(108)
circ_0000520	hsa_circ_0000520	lymph node metastasis, TNM stage, OS	(n=60)	(109)
circHIPK3	hsa_circ_0000284	lymph node metastasis, TNM stage, OS	(n=50)	(114)
hsa_circ_001783	\	tumor size, metastasis, TNM stage, DFS	(n=136)	(115)
circular RNA PVT1	hsa_circ_0001821	TNM stage, OS	(n=99)	(117)
circRAD18	hsa_circ_0002453	tumor size, TNM stage, T stage, OS	TNBC (n=126)	(118)
hsa_circ_0131242	hsa_circ_0131242	tumor size, TNM stage, OS	TNBC (n=86)	(120)
circ_0000291	hsa_circ_0000291	tumor size, lymph node metastasis	(n=37)	(123)
hsa_circRNA_002178	hsa_circ_0000519	OS	(n=70)	(126)
circ-PGAP3	hsa_circ_0106800	tumor size, lymph node metastasis, TNM stage, DFS,OS	TNBC (n=86)	(127)
circIRAK3	hsa_circ_0005505	RFS	(n=122)	(128)
circMYO9B	hsa_circ_0000907	tumor size, lymph node metastasis, TNM stage, OS	(n=21)	(133)
circRNF20	hsa_circ_0087784	tumor size, lymph node metastasis, OS	(n=50)	(134)
circZFR	hsa_circ_0072088	TNM stage, OS	(n=70)	(138)
circ-UBAP2	hsa_circ_0001846	tumor size, lymph node metastasis, distant metastasis, TNM stage, OS	TNBC (n=78)	(139)
circFBXL5	hsa_circ_0125597	OS	(n=150)	(140)
circCNOT2	\	PFS	(n=84)	(144)
hsa_circ_001569	\	lymph node metastasis, clinical stage, OS	(n=75)	(148)
circBACH2	hsa_circ_0001627	T stage, N stage, TNM stage	TNBC (n=38)	(149)
circNFIC	hsa_circ_0002018	lymph node metastasis, OS	(n=145)	(165)
circAHNAK1	hsa_circ_0000320	TNM stage, T stage, N stage, DFS, OS	TNBC (n=136)	(170)
hsa_circ_0072309	hsa_circ_0072309	tumor size, lymph node metastasis, TNM stage, OS	(n=32)	(171)
circRNA_103809	\	distant metastasis, TNM stage, OS, HER2 status	(n=65)	(172)
circDDX17	hsa_circ_0002211	lymph node metastasis, TNM stage	\	(173)
hsa_circ_0068033	hsa_circ_0068033	tumor size, lymph node metastasis, diagnosis	(n=36)	(174)
circ-LARP4	\	tumor size, TNM stage, T stage, N stage, DFS, OS	(n=283)	(178)
circUSP42	hsa_circ_0007823	lymph node metastasis, TNM stage, DFS, OS	TNBC (n=30)	(181)

TNM, tumor node metastasis; DFS, disease free survival; PFS, progression free survival; OS, overall survival; *n = 78 for the study of relationships between circIFI30 expression and clinical stage, histological grade and OS, while n = 38 for the study of the diagnostic value of circIFI30.

As an example of this role, the analyses of several circRNAs can distinguish breast cancer tissues from noncancerous tissues. These distinctions have been shown to occur with favorable statistics, including having area under the curve (AUC) values greater than 0.7. These diagnostic circRNAs include the circular RNA VRK1 (AUC: 0.720, n = 350) (179), circIFI30 (AUC: 0.733, n = 38) (87), circSEPT9 (AUC: 0.711, n = 60) (32) and circAGFG1 (AUC: 0.767, n = 40) (54). In addition, the combination of hsa_circ_006054, hsa_circ_100219, and hsa_circ_406697 has proven useful in diagnosis (AUC: 0.82, n = 51) (55). Although these circRNAs have been shown to be useful as tissue markers, to date, only hsa_circ_0001785 in the plasma has been shown to serve as potentially non-invasive biomarker for breast cancer (AUC: 0.771, n = 20; AUC: 0.784, n = 57) (182).

In addition to diagnostic potential, several circRNAs are prognostic biomarkers for breast cancer. For example, the expressions of circPLK1, circKIF4A, circEPSTI1, circGNB1, or circGFRA1 are associated with the tumor size and TNM stage of TNBC, whereas circPLK1, circKIF4A, circEPSTI1, or circGFRA1 are also associated with the lymph node metastasis of TNBC (56, 81, 88, 89). Similarly, high expression of circPLK1, circKIF4A, circEPSTI1, circGNB1, or circGFRA1 is associated with worse disease-free survival and overall survival in TNBC (56, 81, 88, 89).

## Prospectives

Despite the rapid progress of our understanding of circRNA biogenesis and degradation over the last several decades, many important issues remain unresolved. For example, what signals regulate circRNA-specific expression? What are the intracellular or extracellular signals that activate the mechanisms leading to degradation of circRNAs? Why have only a few blood-localized circRNAs been identified as useful diagnostic markers?

Yet another important question involves potential roles of circRNAs in the communication between the tumor cells and the tumor microenvironment? Although many breast cancer-associated dysregulated circRNAs have been discovered and functionally characterized, most related studies have focused only on the roles of circRNAs on the proliferation, migration, invasion, apoptosis, and chemoresistance of tumor cells. However, tumor heterogeneity is a great challenge for therapeutic management, so growing attention has recently been paid to the molecules that mediate communication between tumor cells and cells of the tumor microenvironment, including cancer-associated fibroblasts, tumor-associated macrophages and T cells. Accordingly, further studies regarding the function of circRNA in mediating the dialogs that occur within or between tumor, stromal and immune cells have great future prospects.

Recently, with the development of ribosome nascent-chain complex (RNC) sequencing and ribo-sequencing, several circRNAs with coding potential have been discovered; however, only a few circRNAs with coding potential have been definitively identified, and most of these studies have indicated that the circRNA-encoded peptides, but not the circRNAs themselves, are the functional unit. Given the broad prospects of peptide therapeutics in the pharmaceutical industry, studies on tumor suppressive circRNA-encoded peptides may provide new directions for the development of drugs for breast cancer.

In addition, as noted in this study, different circRNAs can sponge the same miRNA, but these interactions tend to regulate the expression of different targets. These circRNAs, miRNAs, and targets form regulatory networks and thus promote or inhibit the progression of breast cancer. Therefore, the study of the circRNAs, miRNAs, and target networks in different systems is an important direction for future research.

Another important question involves mechanisms leading to the intracellular localizations of circRNAs. By analyzing the distribution of the tumor suppressive and oncogenic circRNAs in breast cancer (**Table 2**, **Table 3**), we noticed that most of the circRNAs with known function are mainly distributed in the cytoplasm. Five circRNAs, with lengths of 241, 318, 435, 473, and 571 bp, are distributed in both the cytoplasm and nucleus, and two circRNAs, of 269 and 342 bp, are mainly distributed in the nucleus. Similarly, at least 22 cytoplasm-localized circRNAs are less than 400 bp, and this phenomenon cannot be explained by transport mechanisms that solely involve the length of the circRNA, indicating that regulation of the localization of circRNAs is more complex than is currently appreciated.

## Conclusion

CircRNAs form a large class of regulators of breast cancer progression. Clinical studies have also indicated that circRNAs are potential biomarkers for breast cancer diagnosis, prognosis, and therapy. However, the regulation network of circRNAs in breast cancer is still incompletely defined, and this uncertainty impedes the clinical exploitation of circRNAs. Future studies on the precise mechanisms of the key circRNA regulators and clinical relevance to breast cancer will further promote the clinical use of circRNAs and/or their relevant products in the management of breast cancer.

## Data Availability Statement

The original contributions presented in the study are included in the article/supplementary material. Further inquiries can be directed to the corresponding authors.

## Author Contributions

JX wrote the manuscript text and prepared the tables. XC revised the manuscript and prepared the figures. CL and XJ provided advice and revised the manuscript. YuS, YaS, FT, and ML critically reviewed the manuscript. All authors contributed to the article and approved the submitted version.

## Funding

The study was financially supported by the National Natural Science Foundation of China (81902651), Jiangsu provincial key research and development program (BE2019621), Research Innovation Program for Graduates of Jiangsu Province (JX10413758, JX10413759).

## Conflict of Interest

The authors declare that the research was conducted in the absence of any commercial or financial relationships that could be construed as potential conflicts of interest.

The reviewer [ZW] declared a shared affiliation, with no collaboration, with the authors to the handling editor at the time of review.

## Abbreviations

ER, estrogen receptor; PR, progesterone receptor; HER2, human epidermal growth factor receptor 2; BC, breast cancer; LA, luminal A; TNBC, triple-negative breast cancer; NMGT, normal mammary gland tissues; BCLM, breast cancer liver metastases; IPSC, induced pluripotent stem cells; HR, hormonal receptor; TNM, topography, lymph node, metastasis; OS, overall survival; DFS, disease free survival; RFS, recurrence-free survival; BCSC, breast cancer stem cell; EMT, epithelial–mesenchymal transition; PTX, paclitaxel; AUC, area under curve; DALI, distal-Alu-long-intron; SRD, structure-mediated RNA decay; RBP, RNA binding proteins; IRES, internal ribosome entry site; AUC, area under curve.
